# Non-atopic Neonatal Thymic Innate Lymphoid Cell Subsets (ILC1, ILC2, and ILC3) Identification and the Modulatory Effect of IgG From Dermatophagoides Pteronyssinus (Derp)-Atopic Individuals

**DOI:** 10.3389/falgy.2021.650235

**Published:** 2021-04-28

**Authors:** Thamires Rodrigues de Sousa, Fábio da Ressureição Sgnotto, Beatriz Oliveira Fagundes, Alberto José da Silva Duarte, Jefferson Russo Victor

**Affiliations:** ^1^Laboratory of Medical Investigation LIM-56, Division of Clinical Dermatology, Medical School, University of São Paulo, São Paulo, Brazil; ^2^Division of Hematology, Medical School, University of São Paulo, São Paulo, Brazil; ^3^Division of Pathology, Medical School, University of São Paulo, São Paulo, Brazil; ^4^Medical School, Universidade Santo Amaro (Unisa), São Paulo, Brazil; ^5^Faculdades Metropolitanas Unidas (FMU), São Paulo, Brazil

**Keywords:** thymus, human, Derp, ILC1, ILC2, ILC3, IgG

## Abstract

Innate lymphoid cells (ILCs) are classified into distinct subsets termed ILC1, ILC2, and ILC3 cells. The existing literature lacks evidence identifying ILCs and their subsets in the human thymus but already demonstrates that they can exert several functions in regulating immune responses. Furthermore, it was already described that IgG's repertoires could modulate lymphocytes' maturation in the human thymus. Here we aimed to identify ILCs subsets in the human thymus and provide insight into the possible modulatory effect of purified IgG on these cells. Thymic tissues were obtained from 12 infants without an allergic background (non-atopic), and a literature-based peripheral ILCs staining protocol was used. Purified IgG was obtained from non-atopic individuals (n-At), atopic individuals reactive to allergens non-related to dust mites (nr-At), and atopic individuals reactive to the mite Dermatophagoides pteronyssinus (Derp-At). As with all tissues in which they have already been detected, thymic ILCs are rare, but we could detect viable ILCs in all tested tissues, which did not occur with the ILC1 subset. ILC2 and ILC3 NKp44+ subsets could be detected in all evaluated thymus, but ILC3 NKp44- subset could not. Next, we observed that Derp-At IgG could induce the expression of ILC2 phenotype, higher levels of IL-13, and lower levels of IL-4 when compared to IgG purified from non-atopic or non-related atopic (atopic to allergens excluding dust mites) individuals. These results contribute to the elucidation of human thymic ILCs and corroborate emerging evidence about IgG's premature effect on allergy development-related human lymphocytes' modulation.

## Introduction

ILCs encompass classic cytotoxic natural killer (NK) cells and lymphoid tissue inducer (LTi), and non-cytotoxic ILC populations (1, 2). ILCs can be defined as cell lineage marker-negative (Lin^−^) with a typical lymphoid cell morphology (3–5). The non-cytotoxic ILCs consist of three distinct groups termed ILC1, ILC2, and ILC3 cells (6–8). In humans, ILC3 can also distinguish into two subsets based on NKp44 expression (9). The involvement of ILCs was described in several physiological and pathological mechanisms, including adaptive immunity (10), allergy (11), mucosal immunity (12, 13), lung immunity (14), maternal-fetal compartment (15), and several others (16–24).

Due to its importance in the murine and human immune system, the development of ILCs had been studied. Given that ILC derives from the common lymphoid progenitor (CLP) and adaptive immune system lymphocytes (25), the detection of these cells in the primary human organ responsible for most adaptive lymphocytes' maturation has significant importance.

ILC2 cells can collaborate with allergy induction by producing IL-13, these cells are enriched at mucosal barriers in the lung, and their activation is associated with several allergic disorders (26). In this context, a study has shown increased levels of ILC2s in house dust mite (HDM) allergic rhinitis patients and that epithelial cytokine stimulation-induced the production of IL-5 and IL-13 in PBMCs of those patients (27). The enhanced IL-13 ILC2-derived output was also described in IgE-mediated experimental food allergy (28).

HDM is a predominant source of allergens worldwide (29) as it is estimated that they can sensitize 65 to 130 million people worldwide and 50% of asthmatic patients (30). The main HDM species are the *Dermatophagoides pteronyssinus* (Derp), and the allergens of groups 1 and 2, termed *Derp 1* and *Derp 2*, stand out as responsible for ~80% of the reactivity of specific IgE antibodies (30). For these reasons, we focused our study on the Derp atopic IgG repertoire.

Previous studies in the literature demonstrated that IgG antibodies could interact with lymphocytes precursors in the murine and human thymus, modulating its cytokine production as recently revised (31). Together, these pieces of evidence suggest that atopic and non-atopic individuals' IgG repertoire can differentially modulate the maturation of thymic TCD4, TCD8, iNKT B, and γδT cells modulating or inhibiting the development of allergy (32–42). In those observations, it has become evident that IgG antibodies can interact with a wide range of clonal or conserved receptors expressed in several cell populations.

Because of this broad potential of IgG in modulating thymus-matured cells, we aimed to identify thymic ILC subsets (ILC1, ILC2, and ILC3) and evaluate the possible modulatory effect of IgG on the frequency and cytokine production of ILCs. However, it is noteworthy that in this study, compared to previous studies, we refined the grouping of IgG donors considering a specific allergen as cited above. This refinement was shaped because the mechanism mediating IgG modulatory effect is possibly dependent on idiotypes sets (Specificity) that can be influenced according to individuals' exposition to Ambiental allergens, including Derp (31).

## Methods

### Patient Samples

Thymus tissues were obtained from 12 patients who underwent corrective cardiac surgery at the Hospital do Coração (HCor) in São Paulo, Brazil. The evaluated patients exhibited no signs of immunodeficiency, genetic syndromes, or allergic reactions, and patient age of fewer than seven days was used as an inclusion criterion (patient age, mean±standard error (SE): 3.4 ± 0.54 days). Parental allergic backgrounds were evaluated, and only children with non-atopic mothers were included in this study.

Additionally, blood samples were collected from male and female subjects ranging in age from 20 to 40 years old, previously classified into three groups according to their atopic state. The classification conditions were as follows: (I) non-atopic individuals (n-At): clinically confirmed by medical consultation, without significant IgE-specific titers detected for any tested allergens in an immunoblot assay and without reactivity to any tested allergens in a skin prick test (SPT); (II) atopic non-related to Derp individuals (nr-At): clinically allergic as confirmed by medical consultation, with clinical symptom-related IgE-specific titers detected for at least two of the non-HDM tested allergens in an immunoblot assay, and reactivity to at least two of the non-HDM tested allergens in a skin prick test (SPT); (III) Derp atopic individuals (Derp-At): clinically allergic, as confirmed by medical consultation, with clinical symptom-related IgE-specific titers detected for Derp allergen in an immunoblot assay and reactivity to Derp allergen in a skin prick test (SPT). We excluded volunteers with severe eczema or dermographism or used any of the following within 15 days of the test: antihistamines, glucocorticosteroids, or other systemic drugs that could influence the SPT results. After classification, we prepared IgG pools from tolerant (n-At) and allergic (nr-At and Derp-At) individuals.

Additional information about these individuals is shown in Supplementary Table 1. A different donor provided each thymus sample, and six independent experiments were performed to obtain the presented results. The ethics committees at the HCor and the School of Medicine at USP approved this study (CAAE: 15507613.4.0000.0060).

### SPT, Serum Anti-allergen IgE Determination, and Collection of Blood Samples

SPTs were performed following European standards (43) using an adapted panel of allergens, including a Brazilian allergens profile described in Supplementary Table 1.

According to the manufacturer's instructions, serum-specific IgE antibodies were measured with a multiplex immunoblot assay (EUROLINE Inhalation 2–EUROIMMUN AG, Lubek, Germany). The extracts that were tested are also described in Supplementary Table 1. The strips were incubated with patient sera, washed, and incubated with alkaline phosphatase-conjugated anti-human IgE. After the second washing step, the strips were incubated with the chromogen/substrate solution. The reaction was stopped by washing, and the strips were evaluated with EUROLineScan software (EUROIMMUN, Lubek, Germany) to obtain semiquantitative results.

### Purification of IgG

IgG was purified from the previously-stored pooled serum according to the Melon Gel IgG Spin Purification Kit protocol (Thermo, USA), which according to the manufacturer, ensures the production purities more significant than 90% for human IgG and as was already confirmed in previous studies of our group (36, 38, 40–42). Purified IgG was then collected, sterilized using 0.20-micron filters (Corning, Germany), and stored at −80°C for cell culture experiments. According to the manufacturer's instructions, IgG concentrations were determined using Coomassie Protein Assay Reagent (Pierce, USA). Also, we confirmed that the frequencies of IgG subclasses in all three purified IgG pools were similar by performing an ELISA assay using the IgG Subclass Human ELISA Kit (Thermo Scientific, USA). We also submitted purified IgG samples to anti-allergen IgE detection described above to confirm undetectable IgE levels in all tested pools. Total IgA and IgM levels were analyzed in purified IgG samples according to the specifications of the BINDARID Radial Immunodiffusion Kit (RID–Binding Site, UK), and both isotypes were undetectable in all tested samples.

### Thymic Tissue Dissociation and Cell Isolation

Thymocytes were released from the tissue samples using enzymatic dissociation, as described recently by our group (34–38, 42, 44). The thymus was cut into small fragments and placed in 50-mL propylene conical centrifuge tubes. Next, an enzymatic solution (10 mL) consisting of RPMI medium pre-warmed to 37°C, 0.5 mg/mL collagenase A, 0.02 mg/mL DNase I (Roche Diagnostics, Mannheim, Germany), and 5% fetal bovine serum (FBS) was added, and the sample incubated for 10 min at 37°C under continuous agitation. The digested fragments were homogenized gently and filtered through a plastic sieve with a 70-μM mesh screen (Cell Strainer, BD Falcon, CA, USA) to remove aggregates and stromal material. The resultant cell suspensions were washed with 50 mL of RPMI medium (Gibco–Life Technologies, Grand Island, NY, USA) pre-warmed to 37°C and containing 0.1 mg/mL collagenase A and 0.02 mg/mL DNase I (Roche Diagnostics, Mannheim, Germany). Next, the pelleted cells were resuspended in 50 mL of cold PBS containing 5 mM EDTA (Sigma-Aldrich, Saint Louis, MO, USA), 0.02 mg/mL DNase I, and 5% FBS (Gibco, Life Technologies, USA), followed by centrifugation at 540*g* for 5 min. The pelleted cells were resuspended in RPMI medium, and the low-density fraction was collected following centrifugation through Ficoll-Paque (GE Healthcare BioScience, Uppsala, Sweden) at 540*g* for 20 min. The cells were washed twice with RPMI medium and centrifuged at 540*g* for 10 min.

### Freezing and Thawing of Thymic Cells Suspension

After the washing step, the cells were resuspended in a freezing solution (10% Dimethylsulfoxide [DMSO–Synth, Brazil] + 90% Bovine Fetal Serum), and a maximum concentration of 25 × 10^6^ cells/mL was placed on each cryopreservation tube. The cells were frozen with the help of slow freezing boxes (Freezing Box–Nalgene Nunc) containing isopropyl alcohol (Synth, Brazil) at the bottom stored at −80°C for up to 3 days, and then stored in liquid nitrogen.

For thawing, cryopreservation tubes containing the thymocyte suspension were removed from liquid nitrogen and immediately placed in a water bath at 37°C. Vials were gently twirled for <1 min until just a small bit of ice was observed in the vial. Into a laminar flow hood, vials were wiped with 70% ethanol. Pre-warmed RPMI medium was dropwise into the thawed cells suspension and, after the centrifugation, the supernatant was aseptically decanted without disturbing the cell pellet. Cells were gently resuspended in RPMI 1640 medium containing 10% FC-III (HyClone, Logan, UT, USA), and a small sample was diluted 1:2 in Trypan Blue (Sigma, USA) to evaluate cell viability and perform cell counting using a Neubauer chamber under an optical microscope (Labor Optik, Germany). Only vials that yield thawed cells with viability more significant than 75% were used on cell culture experiments, and cell counting to adjust cell concentration was performed considering only viable cells.

### Cell Culture

1 × 10^6^ viable thymocytes were placed in each well of a 48-well-culture plate (CoStar, USA) along with the purified IgG samples previously described in a concentration of 100 ug and maintained in RPMI 1640 medium containing 10% FBS in a total volume of 400 μL at 37°C in 5% CO2 for incubation for 3 days. In the last 24 h, 10 μg/mL Brefeldin A (Sigma, Israel) was added only to the wells dedicated to evaluating intracellular cytokine production. This protocol was previously standardized using positive (Phorbol 12-myristate 13-acetate—PMA) and negative controls (mock condition), and due to the absence of polyclonal stimulation, we could maintain Brefeldin A for 24 h without decreasing cell viability (32, 34, 35, 37, 38, 42).

### Flow Cytometry

Cell staining was performed to evaluate cell labeling via flow cytometry. For cell viability analysis, the cells were incubated with 0.04 μL of LIVE/DEAD® Fixable Red Dead Cell Stain (ThermoFisher, USA) diluted in 1 mL of 1X PBS. The cells were incubated at room temperature for 30 min while protected from light, washed, and resuspended in 250 μL of 1X PBS. All extracellular analyses were performed using viable cells.

Extracellular staining was performed by adding 1 μg of each antibody to the cells (except the unlabelled tubes). Then, the samples were incubated for 30 min at 4°C while protected from light. After that, 500 μL of 1X PBS solution was added, and the tubes were centrifuged at 400*g* for 5 min. The supernatant was discarded by inverting each tube. Then, 300 μL of 1X PBS was added, followed by fixation in 200 μL of 1% formaldehyde for at least 10 min. Thymocytes were stained with mouse anti-human CD45 (APC-Cy7; clone 2D1), Lin (FITC; CD3: clone SK7; CD16: clone 3G8; CD19: clone SJ25C1; CD20: clone L27; CD14: clone MϕP9; CD56: clone NCAM16.2), CD127 (PerCP-Cy 5.5; clone HIL-7R-M21), CRTH2 (BV421; clone BM16), CD117 (CV650; clone 104D2), NKp44 (Alexa 647; clone p44-8) and CD161 (BV510; clone DX12), all provided by BD Biosciences (USA).

For intracellular determination of cytokines, extracellular stained cells were centrifuged at 400*g* for 5 min, the supernatant discarded and added 1 μL of each mouse anti-human antibody following the list: IFN-γ (4S.B3), anti-IL-4 (8D4-8), anti-IL−13 (JES10-5A2), anti-IL-17 (N49-653) labeled with APC, PE, V450, Alexa Fluor 700 (BD Biosciences, USA) and anti-IL−22 (22URTI) labeled with PE-Cy7 (eBioscience, USA). Then, 100 μL of 1x PBS containing 0.05% saponin was added, and the tubes stored at 4°C for 30 min, protected from light. Subsequently, 500 μL of PBS 1% was added and centrifuged for 400*g* for 5 min, and the supernatant was discarded. The cells were resuspended in 300 μL of 1x PBS and fixed with 200 μL of 1% formaldehyde.

**Figure 1:**
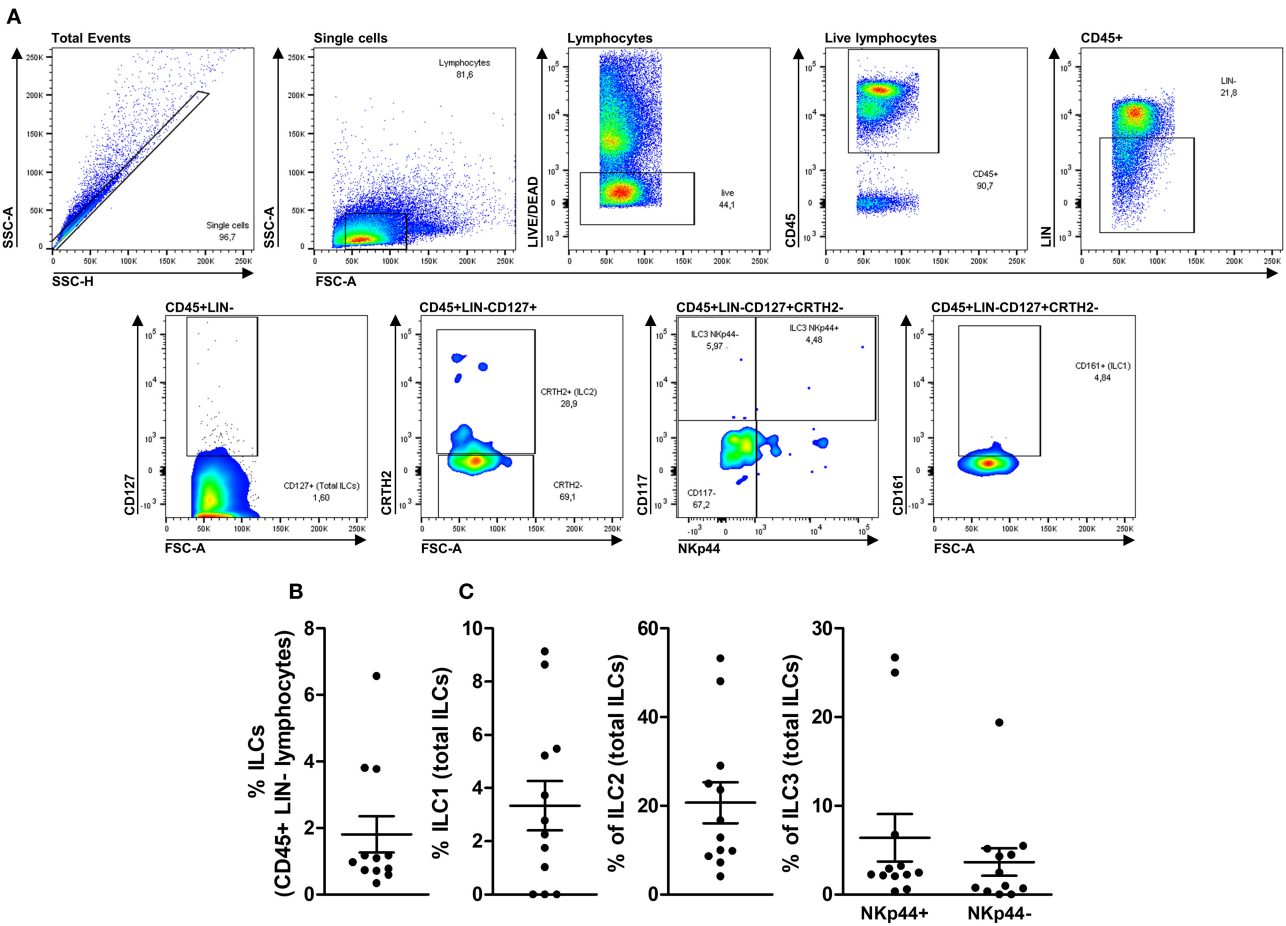
Deep immune-phenotyping of thymic ILCs and their subsets. Upper panels **(A)** illustrate the complete gate strategy to identify total ILCs and their subsets (ILC1, ILC2, ILC3 NKp44+, and ILC3 NKp44-). Thymocytes from 12 children <7 days old were evaluated after thymus dissociation and a brief incubation of 3 days without stimulus. The frequency of total ILCs within CD45+LIN- lymphocytes **(B)** and each ILC subset's rate within total ILCs **(C)** were evaluated by flow cytometry. The symbols represent individual values with mean and standard error.

Using an LSR II Fortessa flow cytometer (BD Biosciences, USA), 500,000 events per sample were acquired in the lymphocyte's quadrant (as determined by their relative size/granularity). Additional information about the equipment configuration is shown in Supplementary Table 2. Compensation was performed using adsorbed microspheres (CompBeads, BD Biosciences, USA) treated with the same antibodies used for extra and intracellular staining. All antibodies were titrated to define 1 μg as an optimal concentration for specific staining. Cell gating was determined using the isotype control values or the fluorochrome minus 1 (FMO) setting to all parameters as illustrated in Supplementary Figures 1, 2. Based on these observations, we determined a gating strategy to identify ILC1, ILC2, ILC3 NKp44+, and ILC3 NKp44- subsets, as demonstrated in Figure 1A. ILCs subsets were identified with the following phenotypes: ILC1 = Single cells with a lymphocyte morphology that were alive and CD45+, LIN-, CD127+, CRTH2-, CD117- and CD161+; ILC2 = Single cells with a lymphocyte morphology that were alive and CD45+, LIN-, CD127+, and CRTH2+; ILC3 = Single cells with a lymphocyte morphology that were alive and CD45+, LIN-, CD127+, CRTH2- and CD117+ with or without NKp44 expression (ILC3 NKp44+ or ILC3 NKp44- cells). As an additional control, we tested the gating strategy in human peripheral cells, as illustrated in Supplementary Figure 3.

### Statistical Analysis

Statistical analysis was performed with GraphPad Prism 5.0 (GraphPad Software Inc., La Jolla, CA). Data from *in vitro* studies were taken from 3 to 5 separate experiments with 12 different thymus donors or 10 n-At, 15 nr-At, or 12 Derp-At individuals. Differences were considered significant at *P* ≤ 0.05, as assessed by one-way ANOVA (Kruskal-Wallis test, comparisons among three groups).

## Results

### Identification of Non-atopic Neonatal Thymic ILCs Subsets

To perform the deep immune-phenotyping of ILCs and their subsets in the human thymus, we consider the previous observation that demonstrates the presence of ILCs in a single human thymus (45) and the deep immunophenotyping of its subsets in a pathological process (46). Furthermore, thymocytes were incubated before staining to re-establish phenotypical properties.

Next, we applied the gating strategy previously described in the Methods section to 12 health neonatal human thymus, and we could observe that total ILCs could be detected in all tested tissues in frequencies ranging from 0.59 to 6.57% of CD45+LIN- cells (Figure 1B). After applying subsets identification gating strategies, we could observe that ILC1s could not be detected in 3 of 12 tested thymi ranging from undetectable to 9.14% of total ILCs. Furthermore, ILC2s could be detected in all evaluated thymus ranging from 4.10 to 53.30% of total ILCs, and, finally, ILC3s NKp44+ could be detected ranging from 0.58 to 26.70% and ILC3s NKp44- ranging from undetectable to 19.40% of total ILCs (Figure 1C).

### Modulatory Effect of Derp-At IgG on Non-atopic Thymic ILCs

**Figure 2:**
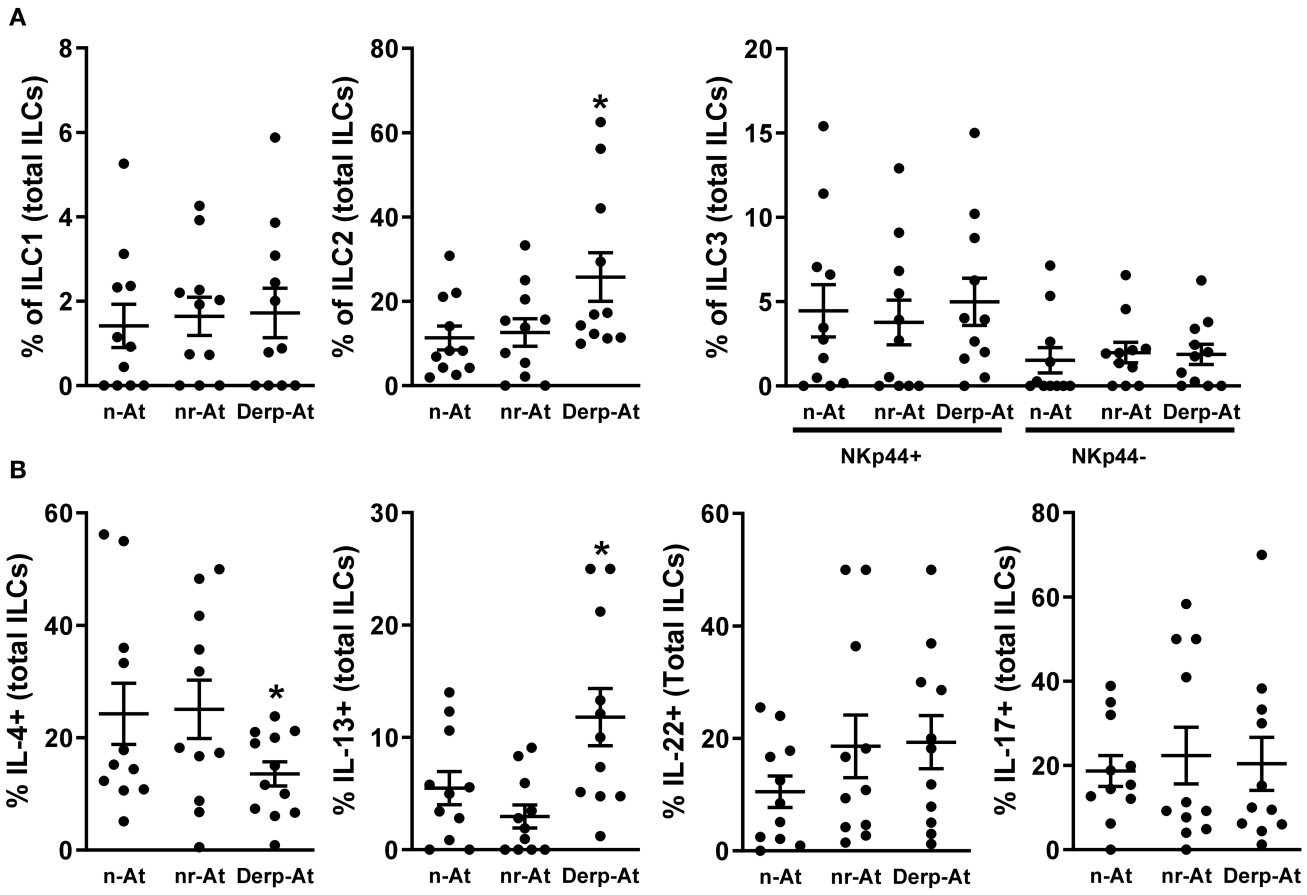
Effects of purified IgG on non-atopic infant intrathymic ILCs. Purified IgG from atopic or non-atopic adults was pooled. Thymocytes from non-atopic infants younger than 7 days old. (*n* = 12) were cultured in the presence of 100 μg/mL of purified IgG from non-atopic (n-At), atopic non-related to Derp (nr-At), or atopic to Derp (Derp-At) individuals. The frequency and viability of each ILC subset within total ILCs **(A)** and the production of IL-4, IL-13, IL-17 e IL-22 by total thymic ILCs **(B)** were evaluated by flow cytometry after 3 days of culture. Data are presented as individual value, mean ± SEM. *p ≤ 0.05 when compared to n-At and nr-At groups.

Non-atopic newborns thymocytes were cultured with purified IgG from the n-AT, nr-At, or Derp-At groups for 3 days. The frequencies and viabilities of ILCs were evaluated and revealed a similar profile between IgG from n-At and nr-At individuals (Figure 2A). In the same experiments, Derp-At's presence on the cultured thymocytes has shown an increased percentage of the ILC2 subset compared to n-At and nr-At conditions suggesting that Derp-At IgG may favor the expression of ILC2 markers in these cells.

Next, we evaluated the production of cytokines on total ILCs, and Derp-At IgG could induce higher levels of IL-13 compared to n-At and nr-At conditions (Figure 2B). Also, Derp-At IgG could be related to lower IL-4 production levels than other conditions (Figure 2B). Because many markers are required for detailed and accurate characterization of ILC, we do not exclude the possibility of comprised cellular contaminations, which could not be excluded due to technical limitations.

## Discussion

The first detailed characterization of ILC in the thymus was suggested by a study utilizing several *in vivo* models (47). However, essential parameters such as cellular viability, leucocytes marker expression (CD45), ILCs subset markers CD161 and NKp44 (45), and sampling to demonstrate an individual's variation were not assessed. These observations demonstrated that the deep immunophenotyping of ILCs in the human thymus yields the detection of ILCs and their subsets but not in all evaluated organs. These results are essential in establishing future approaches to identifying, ontogeny, and isolating ILCs subsets in the human thymus.

In this context, to compare percentages of ILC subsets between our study and the first characterization proposed, it is vital to address that even though there are remarkable anatomical and physiological similarities between humans and animals, particularly mammals, not all results obtained on animals can be directly translated to humans. Even though the characterization previously mentioned was lacking some parameters, and despite the particularities of each study, the percentage of ILC subsets is similar, showing an increase of the ILC2 and lower numbers of ILC3.

The variety of gating strategies that various groups have used to describe these cells' populations, summarizing and comparing the frequencies of ILC subsets described in the literature is quite difficult. Given this situation, a study using a mass cytometry panel analyzed all ILC subsets' frequencies throughout a range of different human tissues such as blood, cord blood, tonsils, spleen, and colon specimen simultaneously. This study has shown that NK cells constituted the main ILC subsets in internal tissues (blood, cord blood, bone marrow, spleen) and lungs. However, in mucosal tissues and skin, ILC2 and ILC3 were more prominent and sometimes outnumbered NK cells (48).

In this study, a critical technical issue was considered and should be highlighted. Due to the low frequency of thymic ILCs, our freezing and thawing protocols were performed very carefully, not allowing thawed cells with viability <75%, an essential aspect for reproducibility between experiments to allow the reliable detection of thymic ILCs.

Apart from that, in the past years, some studies have demonstrated that IgG can play a pivotal role in mediating complex interactions that result in functional lymphocyte modulation during maturation in self or offspring primary lymphoid organs and how this implicates in the context of allergy (33, 37, 49–51). These studies proposed several IgG idiotypes interacting with molecules expressed on B- and T-cell membranes during the maturation process, including clonal receptors (BCRs and TCRs). These interactions result in functional modulation of matures cells, the so-called “Hooks without Bait” theory (31). However, ILCs are a heterogeneous population of lymphocytes that lack antigen-specific receptors (52) but can be activated by cytokines and through natural cytotoxicity receptors (NKp44) (53), so further experiments would be necessary to uncover the mechanism behind the possible modulation of this cell population by IgG.

Between the main HDM species, the literature has presented information regarding different species of mites' role in the immune system's modulation, including a recent study had shown that murine and human allergic asthma in response to *Blomia tropicalis* involves a lymphocyte population (γδT cells) that had not description in Derp-related literature (54). Compilating the suggestion of differential mechanisms regarding the activation of lymphocytes induced by different HDM species, our previous observations of IgG modulatory effect, and our observations of functional human thymic ILCs, we chose to evaluate if IgG can modulate thymic ILCs according to donors' reactivity to the HDM Derp.

Performing this approach in our study, we could observe that Derp-At IgG could favor the ILC2 phenotype and the IL-13 production with a reduction in IL-4 production in non-atopic thymocytes. These observations, combined with the description that ILC2 cells collaborate with allergy induction (27, 55–58), suggest that the IgG repertoire derived from Derp-At individuals had the potential to modulate the maturation of thymic ILCs favoring the acquisition of an allergy-prone profile. Furthermore, the demonstration that the percentage of peripheral ILC2s and the release of IL-13 is elevated in HDM-atopic compared to patients atopic to allergens non-related to HDM (59) reinforces the importance of our findings. Lastly, it was also demonstrated that ILC2 cells increase during the grass pollen season and are inhibited by subcutaneous immunotherapy (60), suggesting some importance to ILC2 cell maturation in the therapeutic context.

In the present study, we did not evaluate IgG membrane binding, but the induction of IgG effects possibly occurs due to membrane interactions. The proposed interactions (IgG-ILCs cell membrane) have not been described in the literature, and several membrane molecules expressed on ILCs can be recognized by IgG in an idiotypic manner and according to IgG idiotypes. The absence of elucidative evidence in this study represents a limitation, but further experiments will be undertaken to elucidate this hypothesis.

Otherwise, consistent with our proposal, the comparison of IgG subclasses from atopic and non-atopic individuals did not reveal differences. Besides, we purified serum IgG from patients without any secondary inflammatory disease for which alterations in peripheral IgG glycosylation have been described (e.g., rheumatoid arthritis or Crohn's disease) (61). After excluding these primary biological differences that can influence the differential modulatory effect of IgG's observed between studied groups, our results corroborate with the literature, indicating that idiotypes can be responsible for the observed effects.

In conclusion, our results demonstrated that ILCs could be identified in neonatal human thymus and that human IgG can modulate cytokines' production by ILC2 cells according to the donors' atopic state. Our pieces of evidence could not elucidate a possible role of IgG-mediated modulation of thymic ILCs. Still, they enrich a recent group of evidence that had been indicated IgG as a potential ligand on the intra-thymic modulation of cells involved in allergy development.

## Data Availability Statement

The original contributions generated for this study are included in the article/Supplementary Material, further inquiries can be directed to the corresponding author/s.

## Ethics Statement

The studies involving human participants were reviewed and approved by The ethics committees at the HCor and the School of Medicine at USP approved this study (CAAE: 15507613.4.0000.0060). Written informed consent to participate in this study was provided by the participants' legal guardian/next of kin.

## Author Contributions

TS: methodology and wrote the manuscript. FS: methodology and validation. BF: methodology. AD: supervision and funding acquisition. JV: funding acquisition, project administration, proposed, and wrote the manuscript. All authors contributed to the article and approved the submitted version.

## Conflict of Interest

The authors declare that the research was conducted in the absence of any commercial or financial relationships that could be construed as a potential conflict of interest.
