# Supplementary material for: Non-atopic Neonatal Thymic Innate Lymphoid Cell Subsets (ILC1, ILC2, and ILC3) Identification and the Modulatory Effect of IgG From Dermatophagoides Pteronyssinus (Derp)-Atopic Individuals
Source: Front Allergy. 2021 Apr 28;2:650235. doi: 10.3389/falgy.2021.650235 (PMC8974683; doi:10.3389/falgy.2021.650235)
Supplement: Supplementary file 1 [file Data_Sheet_1.PDF]

# Supplementary Material

## 1 Supplementary Tables

**Supplementary Table 1:** Characteristics of the individuals included in the study.

| | **n-At** | **nr-At** | **Derp-At** |
|---|---|---|---|
| Number | 10 | 15 | 12 |
| Age, years (mean ± SE) | 34,5 ± 19,13 | 53,2 ± 20,91 | 37,4 ± 16,8 |
| Sex (male/female) | 3/7 | 13/2 | 9/3 |
| **IgE-specific reactivity (n/%)** | | | |
| *Dermatophagoides pteronyssinus* | 0/0 | 0/0 | 12/100 |
| *Dermatophagoides farinae* | 0/0 | 0/0 | 0/0 |
| *Aspergillus fumigatus* | 0/0 | 8/53 | 0/0 |
| *Penicillium notatum* | 0/0 | 4/26 | 0/0 |
| *Alternaria alternata* | 0/0 | 3/20 | 0/0 |
| *Canis familiaris* | 0/0 | 5/33 | 0/0 |
| *Felis domesticus* | 0/0 | 2/13 | 0/0 |
| *Cladosporium herbarum* | 0/0 | 1/0 | 0/0 |
| **SPT[1] reactivity (n/%)** | | | |
| *Dermatophagoides pteronyssinus* | 0/0 | 0/0 | 12/100 |
| *Dermatophagoides farinae* | 0/0 | 0/0 | 12/100 |
| *Aspergillus fumigatus* | 0/0 | 4/26 | 9/75 |
| *Penicillium notatum* | 0/0 | 4/26 | 9/75 |
| *Alternaria alternata* | 0/0 | 4/26 | 9/75 |
| *Canis familiaris* | 0/0 | 1/6 | 2/16 |
| *Felis domesticus* | 0/0 | 4/26 | 4/33 |
| *Cladosporium herbarum* | 0/0 | 4/26 | 9/75 |
| **Others (n/%)** | | | |
| Clinically allergic | 0/0 | 15/100 | 12/100 |

[1] SPT - skin prick test; mo – months

**Supplementary Table 2:** Flow cytometer configurations.

| Laser | PMT | Longpass Dichroic Mirror | Bandpass filter | Fluorochrome or Scatter Parameter |
|---|---|---|---|---|
| **Ultra Violet (355nm)** | A | 505 | 530/30 | Indo-1 (Blue) |
| | B | Blank | 450/50 | Indo-1 (Violet), DAPI, Alexa Fluor 350 |
| **Violet (405nm)** | A | 750 | 780/60 | BV785, QDot 800 |
| | B | 685 | 710/50 | BV711, QDot 700, QDot 705 |
| | C | 635 | 670/30 | BV650, QDot 655 |
| | D | 595 | 610/20 | BV605, QDot 605 |
| | E | 505 | 525/50 | BV510, Am Cyan, Alexa 430, Horizon V500, DAPI |
| | F | Blank | 450/50 | BV421, Horizon V450, Pacific Blue |
| **Blue (488nm)** | A | 640 | 695/40 | PerCP-Cy5.5 |
| | B | 505 | 525/50 | FITC |
| | C | Blank | 488/10 | SSC |
| **Red (640nm)** | A | 750 | 780/60 | APC-Cy7, APC-H7 |
| | B | 710 | 730/45 | Alexa Fluor 700 |
| | C | Blank | 670/14 | APC, Alexa 647 |
| **Yellow/Green (561)** | A | 750 | 780/60 | PE-Cy7 |
| | B | 685 | 710/50 | PerCP-Cy5.5 |
| | C | 635 | 670/30 | PE-Cy5 |
| | D | 600 | 610/20 | PE-CF594, PE-Texas Red, PE-mCherry |
| | E | Blank | 586/15 | PE |

## 2 Supplementary Figures

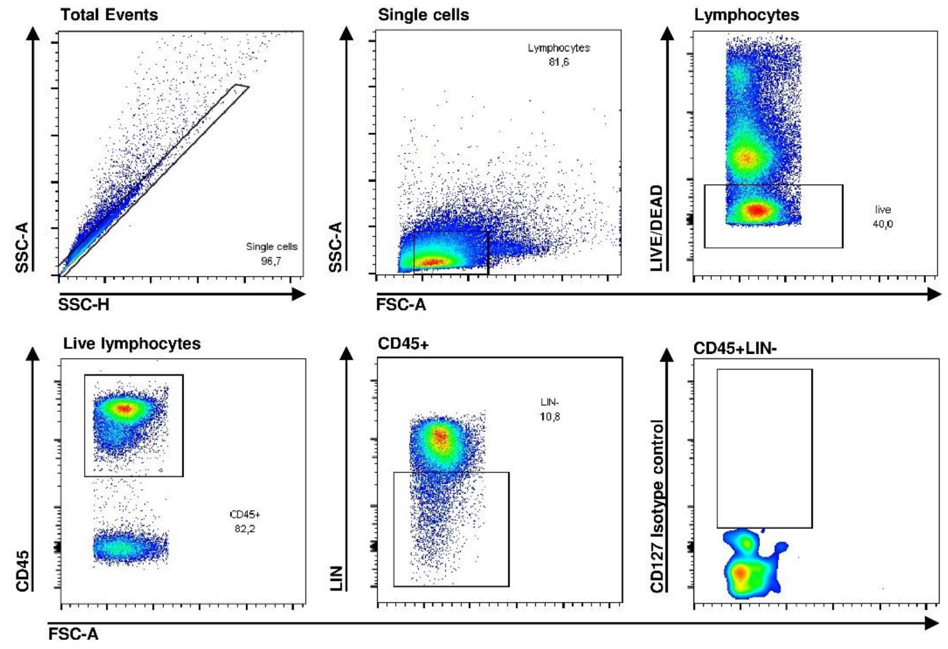


**Supplementary Figure 1: Total ILCs gating strategy illustration.** Panels illustrate the FMO gating strategy to define total ILCs gating. Thymocytes from children less than 7 days old were evaluated after thymus dissociation and a brief incubation of 3 days without stimulus. Cells were stained with LIVE/DEAD, anti-CD45 antibodies, and LIN antibodies cocktail in the presence of anti-CD127 isotype control. Using this protocol, we could define the precise gating of CD45+LIN-CD127+ to identify total ILCs in the fully stained thymus and PBMC samples.

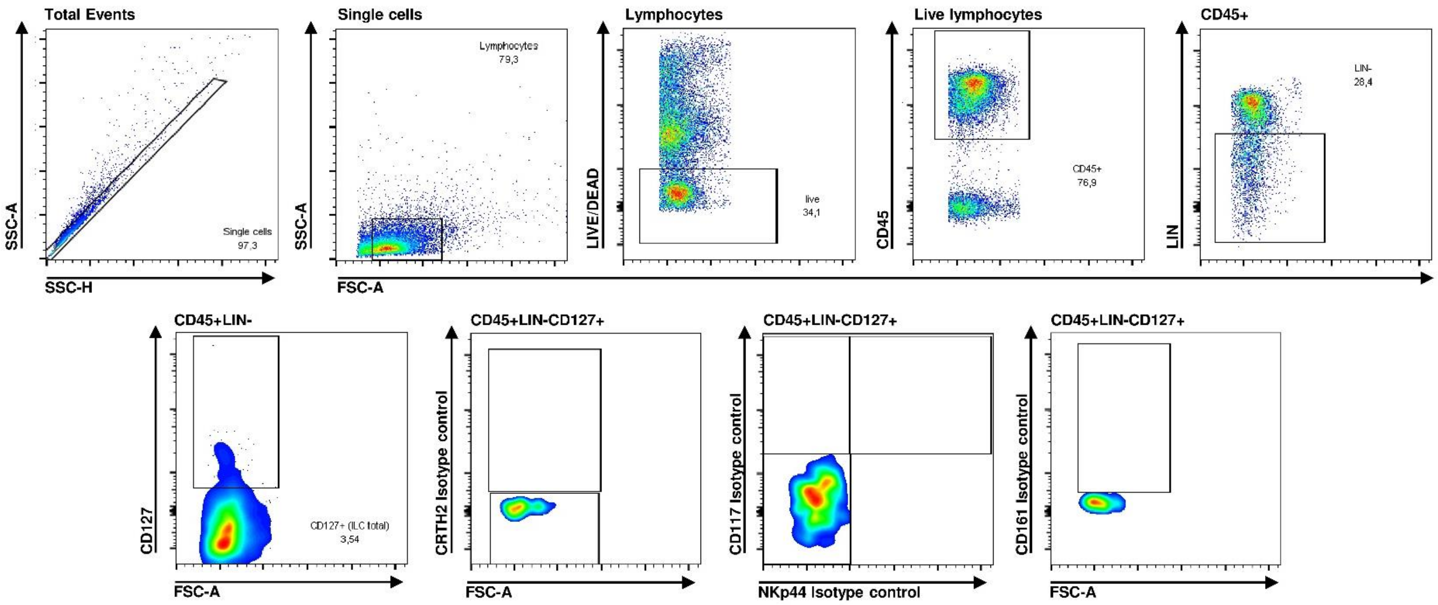


**Supplementary Figure 2: ILCs subsets gating strategy illustration.** Panels illustrate the gating strategy to define each ILC subset gating. Thymocytes from children less than 7 days old were evaluated after thymus dissociation and a brief incubation of 3 days without stimulus. Cells were stained with LIVE/DEAD, anti-CD45 antibodies, LIN antibodies cocktail, and anti-CD127 antibodies in the presence of anti-CRTH2, anti-CD117, anti-NKp44, and anti-CD161 isotype controls. The expression of all isotype controls was evaluated in CD45+LIN-CD127+ cells (Total ILCs). Using this protocol, we could define the precise gating of ILC1 (CD45+, LIN-, CD127+, CRTH2-, CD117- and CD161+), ILC2 (CD45+, LIN-, CD127+, and CRTH2+) and ILC3 (CD45+, LIN-, CD127+, CRTH2- and CD117+ with or without NKp44 expression) cells. The gating strategy defined by this experiment was applied to identify ILCs subsets in the fully stained thymus and PBMC samples.

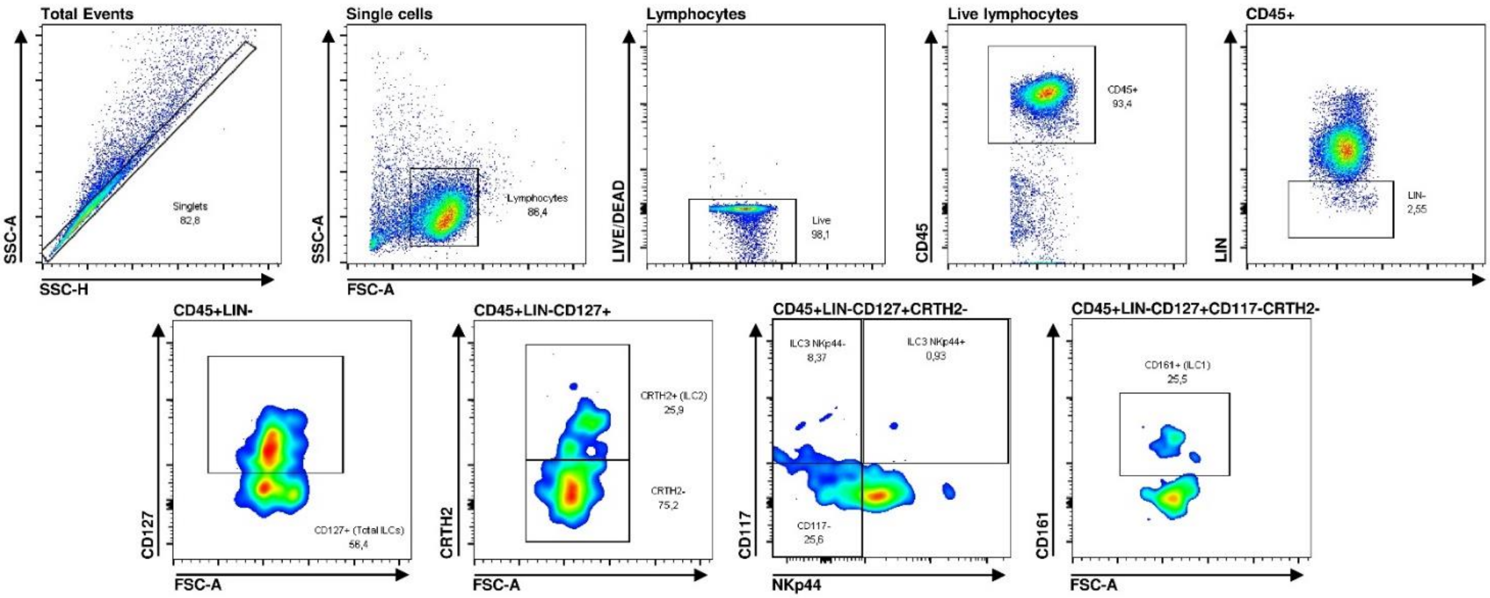


**Supplementary Figure 3: Deep immune-phenotyping of peripheral (PBMCs) ILCs and its subsets.** Panels illustrate the complete gate strategy to identify total ILCs and its subsets (ILC1, ILC2, ILC3 NKp44+, and ILC3 NKp44-). PBMC from a healthy individual were evaluated ex-vivo. We evaluated the frequency of total ILCs (1.35% of CD45+ LIN- lymphocytes) and each ILC subset's rate within total ILCs (ILC1: 4.90%; ILC2: 25.9%; ILC3 NKp44+: 0.70%; ILC3 NKp44+: 6.29%) were evaluated by flow cytometry.
